# Supplementary material for: Dynamic enhancer partitioning instructs activation of a growth-related gene during exit from naïve pluripotency
Source: eLife. 2019 Apr 16;8:e44057. doi: 10.7554/eLife.44057 (PMC6488298; doi:10.7554/eLife.44057)
Supplement: Supplementary file 1. [file elife-44057-supp1.docx]

| **Supplementary File 1. List of primers used in this study** | | | |
| --- | --- | --- | --- |
|  |  |  |  |
| **Genotyping** | **Sequence** | **Notes** |  |
| ∆E3 Fwd | AGGGTGGTGGCTAGAAGACA | Greenberg et al., 2017 |  |
| ∆E3 Rev | GCTAAACTAGCCCCACCAGA | Greenberg et al., 2017 |  |
| ∆E1-3 Fwd ∆ | GCAGACTTCCATCGTGCCCGC |  |  |
| ∆E1-3 Fwd WT | AGGGTGGTGGCTAGAAGACA | Not present in deletion |  |
| ∆E1-3 Rev | GCTAAACTAGCCCCACCAGA |  |  |
| ∆E4 Fwd | AGGCTGTTCAGAGAAAGCCC |  |  |
| ∆E4 Rev | TACATTCTCTCCCACCGGGT |  |  |
| ∆CTCF_PS Fwd | GACAGATGGGAGACAAAGGGG |  |  |
| ∆CTCF_PS_Rev | ATGCTCAGCCGTTAAGAGCA |  |  |
| EedKO Fwd | AGAGTCGAGTCGAGGATGACA | Greenberg et al., 2017 |  |
| EedKO Rev | AGCTGAGCCTTGGCTAACTG | Greenberg et al., 2017 |  |
|  |  |  |  |
| **CRISPR cloning** | **Sequence** | **Notes - All for cloning into pX459** |  |
| ∆E3_L | CACCGATTGGGTGAAGCTTTGGCGT |  |  |
| ∆E3_L | AAACACGCCAAAGCTTCACCCAATC |  |  |
| ∆E3_R | CACCGCAGTTAGGTGACAGTATCCC |  |  |
| ∆E3_R | AAACGGGATACTGTCACCTAACTGC |  |  |
| ∆E1-3_L | CACCGCAGTTAGGTGACAGTATCCC |  |  |
| ∆E1-3_L | AAACGGGATACTGTCACCTAACTGC |  |  |
| ∆E1-3_R | CACCGAAATATGATGTCAGCGTATC |  |  |
| ∆E1-3_R | AAACGATACGCTGACATCATATTTC |  |  |
| ∆E4_L | CACCGTAATTGGCGGTCCGTGCCTG |  |  |
| ∆E4_L | AAACCAGGCACGGACCGCCAATTAC |  |  |
| ∆E4_R | CACCGTACTGGGAATGGGTTCGTAA |  |  |
| ∆E4_R | AAACTTACGAACCCATTCCCAGTAC |  |  |
| ∆CTCF_PS_L | CACCGCCCCAGAGGGTCTTCCCTAG |  |  |
| ∆CTCF_PS_L | AAACCTAGGGAAGACCCTCTGGGGC |  |  |
| ∆CTCF_PS_R | CACCGCCTAGTGACTATGAAATACG |  |  |
| ∆CTCF_PS_R | AAACCGTATTTCATAGTCACTAGGC |  |  |
| Eed_L | CACCGAATAATTGTTAGAAGTGAA | Greenberg et al., 2017 |  |
| Eed_L | AAACTTCACTTCTAACAATTATTC | Greenberg et al., 2017 |  |
| Eed_R | CACCGCCTTGAGTGTACTAGGCTAT | Greenberg et al., 2017 |  |
| Eed-R | AAACATAGCCTAGTACACTCAAGGC | Greenberg et al., 2017 |  |
|  |  |  |  |
| **RT-qPCR** | **Sequence** | **Notes** |  |
| Liz Ex1 Fwd | CGGTGAACATCCCATGCCTT | Greenberg et al., 2017 |  |
| Liz Ex1 Rev | TGACGCCACAACTTGACTGT | Greenberg et al., 2017 |  |
| Zdbf2 Fwd | CCGGAAGGAGAGCAGGAG | Greenberg et al., 2017 |  |
| Zdbf2 Rev | GAGCAGAAAAGAGCAAGCA | Greenberg et al., 2017 |  |
| Fgf5 Fwd | GGGATTGTAGGAATACGAGGAGTT | Buecker et al., 2014 |  |
| Fgf5 Rev | TGGCACTTGCATGGAGTTT | Buecker et al., 2014 |  |
| Otx2 Fwd | CCACTTCGGGTATGGACTTG | Buecker et al., 2014 |  |
| Otx2 Rev | GTCCTCTCCCTTCGCTGTTT | Buecker et al., 2014 |  |
| Gpr1 Fwd | TTCAAACACCACGACAGCTT |  |  |
| Gpr1 Rev | TCCTTTGAGACTTCCATGATG |  |  |
| Eef1b2 Fwd | CCTGTGTCATGCCCTACGTT |  |  |
| Eef1b2 Rev | ACTGCTAGGGCCATACTTGC |  |  |
| Ndufs1 Fwd | CTGTAGGGGCCCTAACCTCT |  |  |
| Ndufs Rev | TGTTACTTCCCACTGCATCCA |  |  |
| Adam23 Fwd | AGGATTCCAGGGTGGCTCTA |  |  |
| Adam23 Rev | TCATCAGTCAGTTCCAGCGG |  |  |
| Rplp2 Fwd | TCCAGAGGCACCATTGAAATT | Nora et al., 2012 |  |
| Rplp2 Rev | TCGCTGGCTCCCACCTT | Nora et al., 2012 |  |
| Rrm2 Fwd | CCGAGCTGGAAAGTAAAGCG | Nora et al., 2012 |  |
| Rrm2 Rev | ATGGGAAAGACAACGAAGCG | Nora et al., 2012 |  |
|  |  |  |  |
| **ChIP-qPCR** | **Sequence** | **Notes** |  |
| E1 Fwd | GTGGCTCTTCGGCTGAGATT |  |  |
| E1 Rev | GGGGTTGCCTAGCTTCCTTT |  |  |
| E2 Fwd | ACACAGTGCCTGGAAGCTAC |  |  |
| E2 Rev | TGCGTTCAACAACTCCCCTT |  |  |
| E3 Fwd | TCGGAAATGTAGGGAGCAGC |  |  |
| E3 Rev | CTCCCAGGCAAGTGAGTCAG |  |  |
| E4 Fwd | GGTTTCAACCGGTTTGAGCC |  |  |
| E4 Rev | GAAAAGGGGCCTCTGATGCT |  |  |
| E Jarid2 Fwd | TAGAGGAGGATCCCGAAACC | Buecker et al., 2014 |  |
| E Jarid2 Rev | TTGGCCTTTGTAAGGTCAGG | Buecker et al., 2014 |  |
| E Fgf5 Fwd | TGGTCTTAGGCAAGGGATTATG | Buecker et al., 2014 |  |
| E Fgf5 Rev | GACAGCAAGCCAATGTGAGA | Buecker et al., 2014 |  |
| pPax5 Fwd | ATGGGAGTTTGTTTTCCTGTGT | Duffie et al., 2014 |  |
| pPax5 Rev | AGTGATGTTTGGCCTAATCCTG | Duffie et al., 2014 |  |
| pLiz Fwd | CAGACAAAGGCCACAGTCAA | Duffie et al., 2014 |  |
| pLiz Rev | GTTTATCGTGAAGGCCGAAA | Duffie et al., 2014 |  |
| -5kb E4 Fwd | TGCTGCTCAGTCATGAAGCA |  |  |
| -5kb E4 Rev | TGGCTGTGTTGCTGAGAACA |  |  |
| -1kb E4 Fwd | GGTGGGTGAACAGCTCAGTT |  |  |
| -1kb E4 Rev | GGGGACTCTTTTGCACCACA |  |  |
| sDMR_A Fwd | TGCTTGCTGGCTCAGTGTAA | Greenberg et al., 2017 |  |
| sDMR_A Rev | ATCTTGCTTCAGCTGGGGAC | Greenberg et al., 2017 |  |
| sDMR_B Fwd | ACTGGTGGGGTTTTGGAGAA | Greenberg et al., 2017 |  |
| sDMR_B Rev | GCCACCACAGGACAAACGTA | Greenberg et al., 2017 |  |
| sDMR_C Fwd | AGTGCACATGCTAGGACACA | Greenberg et al., 2017 |  |
| sDMR_C Rev | ATGAGTGTGCCACTGAACGT | Greenberg et al., 2017 |  |
| pZdbf2 Fwd | CTTTAAGAGCGGGAGGAAGG | Duffie et al., 2014 |  |
| pZdbf2 Rev | AACACTGGGCCCCTTCTG | Duffie et al., 2014 |  |
| pOct4 Fwd | GGCTCTCCAGAGGATGGCTGAG | Greenberg et al., 2017 |  |
| pOct4 Rev | TCGGATGCCCCATCGCA | Greenberg et al., 2017 |  |
| Ctsh Fwd | CAGGGCTGAGACAGAAGGAC | Ferrari et al., 2014 |  |
| Ctsh Rev | CCTGAGAGACCAGAGCCATC | Ferrari et al., 2014 |  |
| CTCF_Left 1 Fwd | CAGAGAAAGGGCCCTGTCAG |  |  |
| CTCF_Left 1 Rev | TCCAGAATCCCCACGCACTA |  |  |
| CTCF_Left 2 Fwd | CAGTATCAGAACGGCCACCA |  |  |
| CTCF_Left 2 Rev | TGCTTGGCTATAAAAGGGAGG |  |  |
| CTCF_PS Fwd | CTTGCTGCTCTTTCAGGGGA |  |  |
| CTCF_PS Rev | CTTCTGTGAATGTGCACGGC |  |  |
| CTCF_H19 Fwd | TGGGCCACGATATATAGGAGTATGCT | Ling et al., Science 2006 |  |
| CTCF_H19 Rev | GAGGTTGGAACACTTGTGTTTCTGGAG | Ling et al., Science 2006 |  |
| CTCF_Hoxa10 Fwd | CGGGCAGAGAAAGAGCAAGA |  |  |
| CTCF_Hoxa10 Rev | TGCATATTTGGAATGCGCCG |  |  |
|  |  |  |  |
| **Pyrosequencing** | **Sequence** | **Notes** |  |
| pLiz Fwd | AGGGTTATATTGAGAGAAATATTGTG | Padmanabhan, Cell 2013 |  |
| pLiz Rev | [Bio]-ATATTAAATTAAACCCTAAATTCCATTTCT | Padmanabhan, Cell 2013 |  |
| pLiz Seq | ATTTAAATTATTGATGTTTAAG | Padmanabhan, Cell 2013 |  |
| sDMR Fwd | AGATGGAAGGAAAGAATTGAATTTTATA | Greenberg et al., 2017 |  |
| sDMR Rev | [Bio]-TACCAACCCACTATATACATTCTTCATTA | Greenberg et al., 2017 |  |
| sDMR Seq | TGAATTTTATATATTATTTTTTGAT | Greenberg et al., 2017 |  |
|  |  |  |  |
| **4C-Seq** | **Sequence** | **Notes** |  |
| pLiz VP iF | AATGATACGGCGACCACCGAGATCTACACTCTTTCCCTACACGACGCTCTTCCGATCTCAAAATAATATCCTCAAATAAGCTCTC |  |  |
| pLIz VP iR_I1 | CAAGCAGAAGACGGCATACGAGATCGTGATGTGACTGGAGTTCAGACGTGTGCTCTTCCGATCTGTATATATTCTAACAAAAGAACATCCTACC | TruSeq Index 1 |  |
| pLIz VP iR_I2 | CAAGCAGAAGACGGCATACGAGATACATCGGTGACTGGAGTTCAGACGTGTGCTCTTCCGATCTGTATATATTCTAACAAAAGAACATCCTACC | TruSeq Index 2 |  |
| CTCF_PS VP iF | AATGATACGGCGACCACCGAGATCTACACTCTTTCCCTACACGACGCTCTTCCGATCTGTTAAGTCTTTGTGTATTGGACATAG |  |  |
| CTCF_PS VP iR_I1 | CAAGCAGAAGACGGCATACGAGATCGTGATGTGACTGGAGTTCAGACGTGTGCTCTTCCGATCTTCCAGAAAAGGGTGTCAAATCTCAT | TruSeq Index 1 |  |
| CTCF_PS VP iR_I2 | CAAGCAGAAGACGGCATACGAGATACATCGGTGACTGGAGTTCAGACGTGTGCTCTTCCGATCTTCCAGAAAAGGGTGTCAAATCTCAT | TruSeq Index 2 |  |
| pZdbf2 VP iF | AATGATACGGCGACCACCGAGATCTACACTCTTTCCCTACACGACGCTCTTCCGATCTGAGTGTGTGCGTGTTACCGA |  |  |
| pZdbf2 VP iR_I1 | CAAGCAGAAGACGGCATACGAGATCGTGATGTGACTGGAGTTCAGACGTGTGCTCTTCCGATCTAAATGGCTCGCGAAGCAACA | TruSeq Index 1 |  |
| pZdbf2 VP iR_I2 | CAAGCAGAAGACGGCATACGAGATACATCGGTGACTGGAGTTCAGACGTGTGCTCTTCCGATCTAAATGGCTCGCGAAGCAACA | TruSeq Index 2 |  |
| Adam23 VP iF | AATGATACGGCGACCACCGAGATCTACACTCTTTCCCTACACGACGCTCTTCCGATCTGCTATCTTCTGACTACGTGG |  |  |
| Adam23 VP iR_I1 | CAAGCAGAAGACGGCATACGAGATCGTGATGTGACTGGAGTTCAGACGTGTGCTCTTCCGATCTCTCAGATTCAGCACCTAGTG | TruSeq Index 1 |  |
| Adam23 VP iR_I2 | CAAGCAGAAGACGGCATACGAGATACATCGGTGACTGGAGTTCAGACGTGTGCTCTTCCGATCTCTCAGATTCAGCACCTAGTG | TruSeq Index 2 |  |
| Nrp2 VP iF | AATGATACGGCGACCACCGAGATCTACACTCTTTCCCTACACGACGCTCTTCCGATCTTTCTCCAAATGCTCTCTCAC |  |  |
| Nrp2 VP iR_I1 | CAAGCAGAAGACGGCATACGAGATCGTGATGTGACTGGAGTTCAGACGTGTGCTCTTCCGATCTTGGGAAATACAGCACTGAAA | TruSeq Index 1 |  |
| Nrp2 VP iR_I2 | CAAGCAGAAGACGGCATACGAGATACATCGGTGACTGGAGTTCAGACGTGTGCTCTTCCGATCTTGGGAAATACAGCACTGAAA | TruSeq Index 2 |  |
